# Regulatory and HTA framework for herbal medicines in Indonesia

**DOI:** 10.1080/20523211.2026.2640252

**Published:** 2026-03-16

**Authors:** Mohamad Kashuri, Taruna Ikrar, Rani Sauriasari, Vishnu Juwono, Arry Yanuar

**Affiliations:** a Faculty of Pharmacy, Universitas Indonesia, Depok, Indonesia; b The Indonesian Food and Drug Authority, Jakarta, Indonesia; c Faculty of Administrative Science, Universitas Indonesia, Depok, Indonesia

**Keywords:** Evidence generation, health technology assessment, herbal medicines, pharmacovigilance, regulatory framework

## Abstract

**Background:**

Herbal medicines are widely used in Indonesia and other LMICs, yet their integration into national health systems relies on coherent regulatory oversight, reliable evidence generation, functional pharmacovigilance (PV), and alignment with health technology assessment (HTA) and financing processes. Existing evidence varies in rigour and maturity, creating uncertainty for regulators and policymakers. A consolidated understanding of how available evidence informs regulatory, HTA, PV, and financing decisions is needed to guide a more predictable, evidence-informed governance framework for herbal medicines in Indonesia.

**Methods:**

An integrative review was conducted using Scopus, PubMed, Google Scholar, WHO IRIS, the ASEAN TMHS repository, and national regulatory portals for literature published between 2015 and 2025. Thirty-seven studies met eligibility and WHO-based methodological quality criteria. Data were extracted using a structured matrix, synthesised thematically, and deductively mapped to five analytic domains: evidence generation, regulatory pathways, HTA processes, pharmacovigilance systems, and financing–governance alignment.

**Results:**

Three system-level themes emerged. First, substantial misalignment exists between available evidence and regulatory requirements, driven by heterogeneous clinical methods, limited comparator data, and variable standardisation. Second, HTA remains weakly integrated into decision-making due to gaps in clinical effectiveness, limited economic evidence, and challenges in assessing multi-component interventions. Third, PV and governance systems show fragmentation, weak inter-agency coordination, and inadequate safety signal detection. These interdependent weaknesses reduce regulatory predictability, constrain HTA feasibility, and limit informed financing decisions.

**Conclusion:**

This review provides the first integrated synthesis of evidence generation, regulatory pathways, HTA processes, PV systems, and financing–governance alignment for herbal medicines in Indonesia. Findings highlight the need to strengthen evidence standards, adapt HTA methodologies, reinforce PV and laboratory capacity, and improve regulatory–financing linkages. Implementing the proposed framework can enhance policy coherence, regulatory transparency, and safety oversight, supporting more credible and evidence-informed integration of herbal medicines into Indonesia’s health system.

## Background

Herbal medicines continue to play a significant role in healthcare systems across many low- and middle-income countries (LMICs), including Indonesia, where they are widely used for self-medication, cultural practices, and complementary therapy. Global estimates indicate that up to 80% of populations in Asia and Africa rely on herbal products for primary health needs, underscoring their systemic relevance to population health (WHO, 2019). Beyond LMICs, substantial use of herbal medicines and complementary therapies is also reported in developed countries, where they are routinely used as adjuncts to conventional care for chronic disease management, prevention, and patient-centred wellness, confirming that complementary therapy constitutes a global health phenomenon with clear policy and regulatory implications rather than an LMIC-specific practice (Harris et al., 2012; Lee et al., 2022; Steel et al., 2018). In Indonesia, the expanding use of *jamu*, *obat herbal terstandar* (standardised herbal medicines), and *fitofarmaka* (phytopharmaceuticals) reflects rising consumer demand and national interest in strengthening domestic health resources (The Indonesian Government, 2023). Taken together, these trends necessitate coherent regulatory frameworks, fit-for-purpose health technology assessment (HTA) mechanisms, and evidence-informed policy processes to ensure the quality, safety, and clinical value of herbal medicines within national health systems (OECD, 2020).

The regulatory landscape for herbal medicines varies widely across countries, shaped by national health priorities, differing regulatory maturity, and diverse approaches to evidence generation. Herbal products may be classified as traditional medicines, natural health products, botanical drugs, or dietary supplements, resulting in divergent requirements for safety evaluation, manufacturing standards, and market authorisation (EMA, 2022). Such heterogeneity creates challenges for achieving regulatory consistency, especially within regions characterised by substantial cross-border trade such as ASEAN (2022). While Indonesia’s regulatory authority (BPOM) has made progress in strengthening oversight, persistent system-level constraints remain, including limited standardisation, gaps in post-market surveillance, and weak alignment across regulatory, financing, and HTA subsystems (Mercado et al., 2022).

Evidence-informed policymaking in the pharmaceutical sector relies on robust clinical evidence, reliable safety data, and real-world performance indicators. However, herbal medicines often lack the standardised clinical trial designs, validated outcome measures, and pharmacovigilance (PV) systems required to meet conventional regulatory expectations (Divakar, 2022). Their multi-component nature, natural variability, and context-dependent usage patterns further complicate standardisation and reproducibility (Heinrich et al., 2018). These challenges also limit the applicability of HTA, which depends on comparative effectiveness, utility measures, and cost-effectiveness modelling to inform reimbursement and benefit-package decisions (Nemzoff et al., 2021; Trowman et al., 2023). Consequently, many LMIC policymakers struggle to incorporate herbal medicines into formal health financing systems due to insufficient or inconsistent evidence (Chalkidou et al., 2013; Glassman & Chalkidou, 2012).

WHO has emphasised the importance of strengthening regulatory systems and integrating evidence-based approaches to safeguard the use of traditional and complementary medicines (WHO, 2019). The Global Benchmarking Tool (GBT) identifies regulatory policy, inspection, clinical evaluation, PV capacity, and laboratory infrastructure as essential components of mature regulatory systems (WHO, 2021b). Yet assessments across LMICs consistently reveal gaps in regulatory governance, limited inter-agency coordination, and weak linkages between regulation, HTA, and financing (Kim & Jeon, 2020). These systemic challenges closely mirror the Indonesian context, where herbal medicine governance is shaped by intersecting public health priorities, industrial development objectives, and persistent limitations in evidence generation (Sharma et al., 2021).

Safety concerns also remain central to the governance of herbal products. Reports of adulteration, contamination, substandard manufacturing, and herb–drug interactions have been documented in regional and global literature, underscoring the need for effective PV systems (Posadzki et al., 2013). Underreporting of adverse events further limits risk management capacity in LMICs (Heinrich et al., 2018), reinforcing the need for improved surveillance, laboratory verification, and strengthened reporting culture among health professionals and consumers (Shaw et al., 2012).

From an economic perspective, HTA serves as a key instrument for generating reimbursement recommendations and guiding efficient resource allocation. However, HTA for herbal medicines continues to face methodological constraints, including the absence of standardised comparators, limited utility data, and difficulties evaluating multi-component formulations (Zisis et al., 2024). These constraints are especially relevant to Indonesia’s efforts to strengthen evidence-informed benefit-package design within the national health insurance system, where insufficient economic evidence has restricted the formal inclusion of herbal products in coverage deliberations (Rajsekar, 2018).

Taken together, these challenges illustrate the need for an integrated, systems-based framework that aligns regulatory science, HTA methods, PV functions, and health financing mechanisms. Although the literature highlights the value of multisectoral governance, cross-agency coordination, and harmonised regulatory pathways in enhancing pharmaceutical systems (O’Brien et al., 2021), such approaches remain substantially underdeveloped for herbal medicines due to fragmented institutional roles and uneven technical capacity (Nemzoff et al., 2021). Evidence gaps, regulatory inconsistencies, and surveillance limitations collectively impede predictable, evidence-informed decision-making for herbal medicines in Indonesia. This review focuses specifically on Indonesia while drawing selectively on global and regional evidence to contextualise regulatory and HTA challenges.

This review therefore synthesises the existing evidence base to develop a coherent regulatory and HTA framework tailored to the Indonesian context. Specifically, it aims to (1) map the types and maturity of evidence informing regulatory, HTA, and PV processes; (2) identify system-level gaps across evidence generation, regulatory structures, HTA processes, financing mechanisms, and governance; and (3) propose an evidence-informed framework capable of supporting more consistent, transparent, and integrated oversight of herbal medicines in Indonesia. By employing an integrative review approach and combining inductive thematic analysis with deductive domain mapping, this study provides policy-relevant insights to strengthen Indonesia’s pharmaceutical governance and to support the safe, effective, and value-based integration of herbal medicines.

## Methods

### Study design

This study used an integrative review approach based on the methodology of Whittemore and Knafl (2005), allowing synthesis of heterogeneous evidence sources relevant to herbal medicine governance. This integrative review approach was selected to enable the systematic synthesis of diverse evidence types, including empirical studies, regulatory documents, and policy analyses, which are not adequately captured through conventional systematic reviews focused primarily on clinical effectiveness. The design was selected because regulatory, HTA, pharmacovigilance, and financing questions require incorporation of empirical studies, regulatory documents, and policy analyses evidence types that cannot be captured through conventional clinical systematic reviews. The review followed five stages: problem identification, literature search, data evaluation, data analysis, and integration of findings.

### Problem identification

The review assessed how clinical evidence, safety surveillance data, economic evaluations, and regulatory documents currently inform Indonesia’s regulatory, HTA, and health financing systems for herbal medicines. The guiding questions were: (1) What forms of evidence inform regulatory, HTA, and pharmacovigilance processes?; (2) How effectively are this evidence streams incorporated into decision-making?; and (3) What components are needed for an evidence-informed regulatory and HTA framework suitable for Indonesia’s health system?

### Literature search strategy

A structured search was conducted in Scopus, PubMed, Google Scholar, WHO IRIS, and the ASEAN TMHS repository for publications from 2015–2025. National regulatory and policy documents were retrieved from BPOM and Ministry of Health portals. The search string used was: (‘evidence-based policy’ OR ‘evidence-informed decision’) AND (‘herbal medicine’ OR ‘traditional medicine’ OR ‘phytopharmaceutical’) AND (‘regulation’ OR ‘HTA’ OR ‘pharmacovigilance’). Searches were limited to English and Indonesian. Grey literature was included when it provided methodological, regulatory, or policy-relevant content. All records were managed in Zotero and duplicates removed prior to screening.

### Eligibility criteria

Eligible publications included empirical studies, conceptual analyses, and policy-oriented documents addressing herbal medicine regulation, safety surveillance, evidence generation, HTA, or health financing. Inclusion criteria were: (1) English or Indonesian language; (2) publication years 2015–2025; and (3) relevance to pharmaceutical policy or regulatory decision-making. Exclusion criteria were opinion pieces lacking methodological detail, studies unrelated to regulatory or policy issues, inaccessible full texts, and duplicate records.

### Study selection

**Figure 1.**
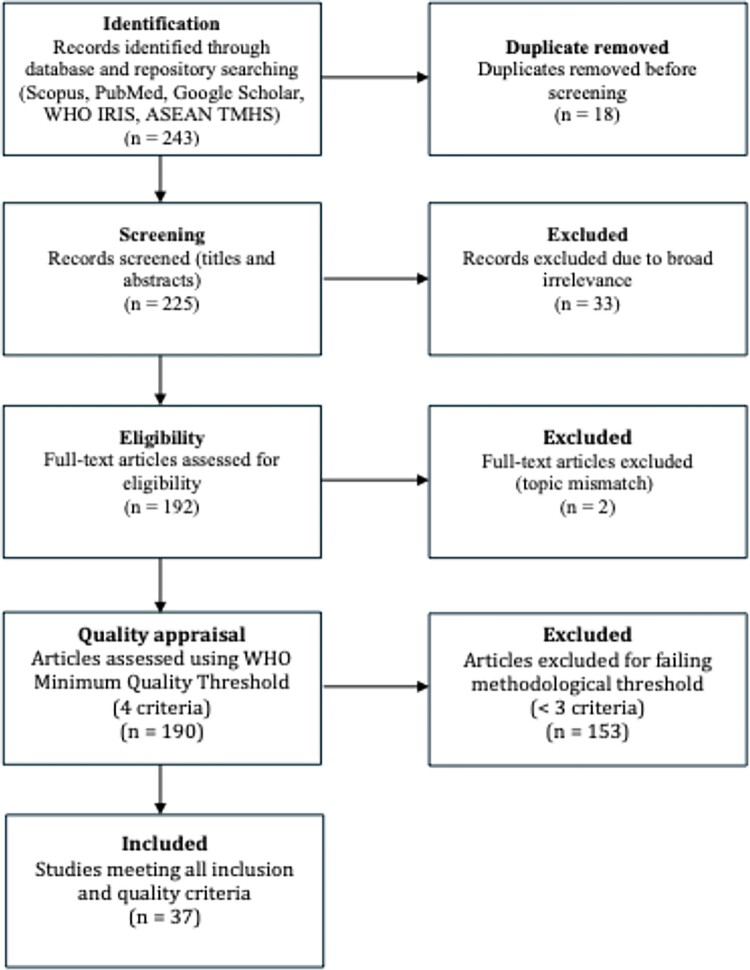
PRISMA 2020 study selection flowchart.

Study selection followed PRISMA 2020 procedures. Titles and abstracts were screened independently by two reviewers, followed by full-text assessment. Any discrepancies in study selection were resolved through discussion and consensus to ensure consistency and transparency in inclusion decisions. Of 243 initial records, 18 duplicates were removed. After screening 225 titles and abstracts, 33 records were excluded for irrelevance. A total of 192 full texts were assessed, with 2 excluded for mismatch in topic. The remaining 190 publications underwent quality appraisal, yielding 37 studies that met inclusion and minimum quality criteria. A summary PRISMA flow diagram is provided in Figure 1.

### Quality appraisal

Methodological quality was evaluated using a simplified WHO-based minimum quality threshold, assessing four criteria: clarity of objectives, methodological adequacy, validity of data sources, and relevance to regulatory or policy decision-making. This quality appraisal approach represents an operational adaptation of WHO principles for the purpose of policy-oriented and regulatory evidence synthesis, rather than a full application of the WHO Global Benchmarking Tool. Each study was appraised independently by reviewers, with disagreements resolved through discussion. Studies meeting fewer than three criteria were excluded. Thirty-seven empirical studies, regulatory analyses, and policy papers met the minimum quality threshold.

### Data extraction

A structured extraction matrix was used to capture publication characteristics, study design, evidence type and maturity, and relevance to regulatory, HTA, or pharmacovigilance domains. Extracted information was mapped to five analytic domains: (1) evidence generation; (2) HTA and economic evaluation; (3) regulatory governance; (4) integration with national health financing; and (5) policy feedback and system alignment. All extracted data were cross-checked for accuracy and completeness.

### Data analysis

Data synthesis combined inductive thematic coding with deductive mapping to the five analytic domains. Descriptive mapping summarised study characteristics and the distribution of evidence. Inductive coding identified recurrent patterns such as evidence gaps, regulatory inconsistencies, HTA limitations, and pharmacovigilance weaknesses. These codes were organised into thematic clusters reflecting system-level relationships across regulation, HTA, surveillance, and financing. The thematic synthesis informed development of the integrated regulatory and HTA framework presented in the Results section. Summary matrices were used to present cross-domain linkages while avoiding narrative redundancy.

### Ethical considerations

This review used only published academic literature and publicly accessible policy documents. As no human subjects or identifiable personal data were involved, ethical approval was not required under ICMJE or JPPP guidelines.

**Figure 2.**
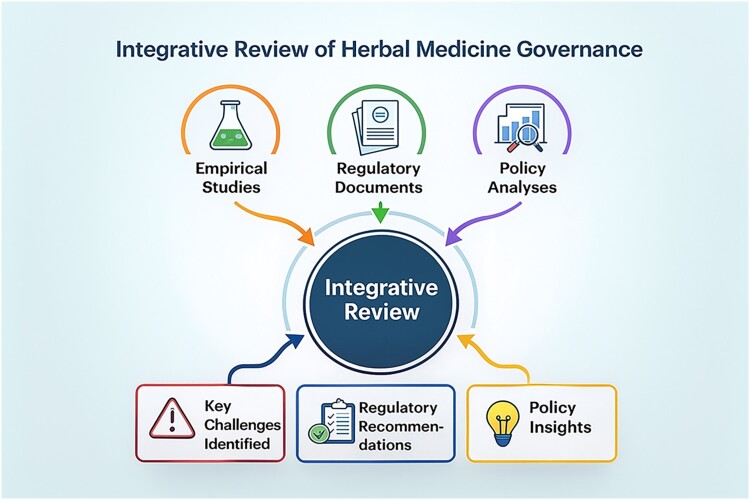
Integrative review framework.

A graphical abstract summarising the overall evidence flow, analytic stages, and outputs of the integrative review is presented in Figure 2.

## Results

A total of 37 studies met all eligibility and methodological quality thresholds and were included in the final synthesis. These publications encompassed clinical studies, regulatory evaluations, pharmacovigilance analyses, economic assessments, and broader policy papers, reflecting the heterogeneous evidence base governing herbal and traditional medicines (Liang et al., 2021; von Schoen-Angerer et al., 2023; WHO, 2022). Using descriptive mapping, inductive coding, and deductive alignment with five analytic domains, the review generated an integrated interpretation of how evidence currently supports Indonesia’s regulatory and HTA systems for herbal medicines.

### Descriptive mapping of evidence types and study characteristics

The included literature varied considerably in methodological approach and policy relevance. Regulatory and governance analyses constituted the largest share (*n* = 18), highlighting inconsistencies in product categorisation, variations in quality standards, and limited regulatory capacity across LMIC contexts. Several studies provided concrete examples of regulatory bottlenecks, including inconsistent classification of herbal products across regulatory categories, unclear minimum evidence requirements for market authorisation, and variability in dossier expectations, which collectively reduced regulatory predictability and prolonged approval timelines. Nine studies focused on pharmacovigilance and safety surveillance, frequently reporting limitations in adverse event reporting systems, passive detection mechanisms, and inadequate laboratory confirmation capacity. These limitations reflected both structural constraints in surveillance system design and operational capacity gaps in implementation. Six studies addressed HTA and economic evaluation, identifying methodological constraints in assessing herbal interventions and insufficient cost-effectiveness data. HTA-specific bottlenecks included the absence of suitable comparators, limited availability of utility measures, and lack of budget impact analyses, restricting the production of decision-relevant HTA outputs. The remaining four studies examined multisectoral coordination, financing arrangements, and integration of herbal products into benefit packages.

Overall, this distribution reflects persistent gaps in clinical, safety, and economic evidence needed to inform regulatory and HTA processes. These gaps are consistent with broader analyses demonstrating that traditional medicine interventions are seldom supported by rigorous comparative trial data or policy-relevant economic modelling (Hogervorst et al., 2022; Skivington et al., 2021). Clinical evidence likewise remains inconsistent, with limitations in trial design, outcome standardisation, and follow-up duration mirroring concerns documented in assessments of herbal medicine research quality (Heinrich et al., 2018). Importantly, the lack of HTA outputs aligned with reimbursement criteria was repeatedly linked to weak or absent financing decisions, limiting the translation of regulatory approval into coverage or benefit-package inclusion. Table 1 presents a summary of study characteristics across evidence types and methodological domains.

**Table 1.** Characteristics of included studies.

Characteristic	Category / Description	Number of Studies (*n*)	Proportion (%)
Study Type	Regulatory / policy analysis	18	48.6%
	Pharmacovigilance & safety studies	9	24.3%
	HTA & economic evaluation	6	16.2%
	Health financing & governance	4	10.8%
Geographic Focus	Indonesia-specific	15	40.5%
	Multi-country LMIC comparisons	12	32.4%
	Global/ non-regional	10	27.1%
Evidence Type	Clinical / therapeutic evidence	11	29.7%
	Safety / pharmacovigilance evidence	14	37.8%
	Economic / HTA evidence	6	16.2%
	Regulatory & governance evidence	25	67.5%
Methodological Approach	Empirical quantitative	9	24.3%
	Empirical qualitative	7	18.9%
	Mixed methods	5	13.5%
	Conceptual / policy analysis	16	43.2%
Quality Score (WHO 4-criteria)	Meets all 4 criteria	19	51.4%
	Meets 3 criteria	18	48.6%
	Meets ≤2 criteria (excluded)	0*	–
Domain Contribution	Regulatory pathways	28	75.7%
	Evidence generation	20	54.0%
	Pharmacovigilance systems	17	45.9%
	HTA processes	12	32.4%
	Financing & reimbursement	9	24.3%

Note: Studies scoring <3 criteria were excluded during quality appraisal.

### Inductive coding: emerging cross-cutting patterns

Four recurring patterns emerged from inductive coding.

First, evidence gaps were prominent, including limited comparator-controlled studies, inconsistent safety reporting, and absence of real-world evidence (Gagnier et al., 2006; Li et al., 2024). The methodological heterogeneity of available studies frequently fell short of regulatory expectations (EMA, 2025).

Second, regulatory inconsistencies were evident, particularly in classification systems, dossier requirements, and quality control standards across countries, especially where regional harmonisation is limited (Liang et al., 2021). These inconsistencies constituted recurrent regulatory bottlenecks that constrained transparent decision-making and reduced alignment between evidence generation and regulatory appraisal.

Third, HTA constraints were reported, with challenges in defining comparators, limited availability of utility measures, heterogeneity in clinical endpoints, and structural difficulties in modelling multi-component traditional formulations (Chen, 2022). These HTA bottlenecks directly affected financing pathways, as insufficient HTA evidence limited the feasibility of reimbursement decisions and weakened the linkage between regulatory authorisation and health financing mechanisms.

Fourth, pharmacovigilance weaknesses were identified, including underreporting, minimal signal detection capacity, and fragmented interfaces between healthcare providers, laboratories, and regulatory authorities concerns also highlighted in WHO assessments of traditional medicine surveillance systems (WHO, 2021b). The review distinguishes structural PV gaps, such as fragmented institutional mandates and weak system integration, from capacity-related gaps, including limited human resources, inadequate laboratory testing capacity, and low reporting rates of adverse events.

### Deductive mapping to five analytic domains

The inductive themes were then mapped against the five analytic domains defined in the Methods. Gaps in evidence generation were found to weaken both regulatory appraisal and HTA feasibility, as insufficient clinical, safety, or economic data undermine transparent decision-making. Regulatory inconsistencies were linked to variability in dossier expectations and difficulties applying evidence in classification and authorisation processes (Liang et al., 2021). HTA limitations were shown to constrain financing decisions by reducing the availability of cost-effectiveness and budget impact evidence required for coverage deliberations, thereby weakening alignment between regulatory approval, HTA outputs, and reimbursement policy (Bloem et al., 2021; Yan et al., 2023). Governance fragmentation, reflected in limited cross-agency coordination, further complicated this landscape and impeded integrated decision-making.

### Thematic clusters: system-level themes

Three overarching themes emerged from thematic clustering.

#### Theme 1. Misalignment between evidence standards and regulatory requirements

Available evidence rarely meets regulatory expectations for safety, quality, and efficacy (Thakkar et al., 2020). Variability in methodologies, comparator selection, and chemical standardisation contributes to this misalignment (Gagnier et al., 2006), echoing broader calls for stronger evidence pipelines to support predictable regulatory decision-making (Burns et al., 2022).

#### Theme 2. Limited integration of HTA in herbal medicine decision-making

HTA uptake remains hindered by gaps in clinical and economic evidence, lack of standardised outcomes, and difficulty modelling multi-component interventions. Weak HTA–financing linkages were evident, as the absence of decision-ready HTA outputs constrained reimbursement decisions and limited the integration of herbal medicines into benefit packages (Downey et al., 2017; MacQuilkan et al., 2018).

#### Theme 3. Weak post-market surveillance and fragmented governance

Studies repeatedly highlighted underreporting of adverse events, passive surveillance, and limited laboratory verification. Structural PV fragmentation and capacity constraints jointly reduced the effectiveness of safety signal detection, regulatory enforcement, and feedback into HTA and financing processes (Garashi et al., 2022; Menang et al., 2023).

Table 2 summarises the cross-domain evidence linkages identified through this synthesis, highlighting how evidence supports regulatory, HTA, safety, and financing functions.

**Table 2.** Cross-domain evidence linkages identified in the integrative review.

Analytic Domain	Core Evidence Characteristics Identified (from 37 studies)	System-Level Function This Evidence Supports	What the Evidence Reveals (Gaps + Opportunities)
1. Evidence Generation	Clinical data often fragmented; safety data variable; limited economic evaluations; real-world evidence scarce.	Establishes foundational knowledge for regulatory classification, minimum evidence standards, and early HTA scoping.	Gaps in standardisation and methodological consistency limit the ability of regulators and HTA bodies to appraise herbal products transparently.
2. Regulatory Pathways	Documentation focuses on quality and safety; limited integration of clinical/economic data; inconsistent dossier expectations for herbal categories.	Supports decision-making for market authorisation and product categorisation.	Evidence shows misalignment between dossier requirements and available evidence maturity, creating regulatory uncertainty for applicants.
3. HTA Processes	Sparse cost-effectiveness analyses; limited budget impact studies; heterogeneous clinical endpoints.	Helps determine reimbursement eligibility and formulary placement.	Evidence reveals that existing HTA tools are not calibrated for multi-component herbal interventions, limiting their use in national coverage decisions.
4. Pharmacovigilance Systems	Variable signal detection; insufficient standardised reporting; limited linkage to regulatory reassessment.	Ensures post-market safety, benefit–risk monitoring, and feedback into regulatory review.	Evidence shows weak surveillance integration and underreporting, preventing timely regulatory action for herbal products.
5. Health Financing & Coverage	Few studies linking herbal use to financial protection, claims data, or cost–effectiveness.	Guides inclusion/exclusion in benefit packages and supports priority-setting.	Evidence highlights lack of clear decision rules and insufficient economic justification for coverage, resulting in fragmented financing policies.

**Figure 3.**
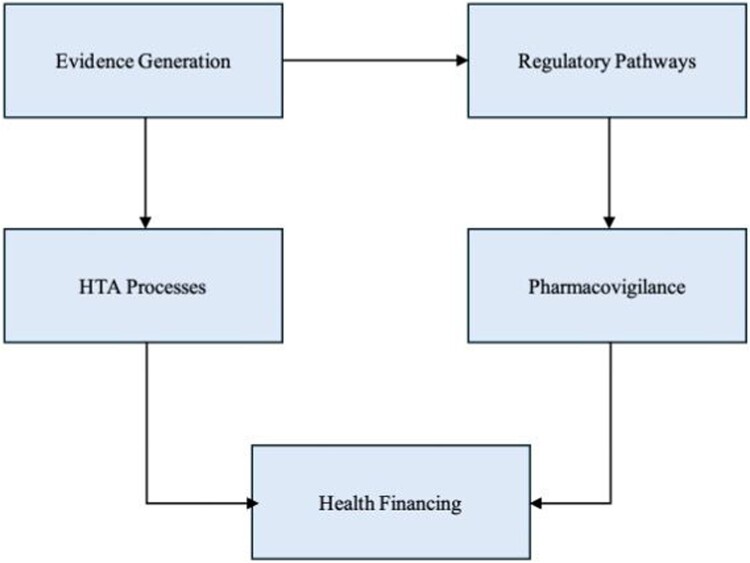
Integrated regulatory–HTA framework for herbal medicines.

These patterns collectively demonstrate that regulatory, HTA, surveillance, and financing functions are interdependent, and that weaknesses in one domain generate constraints across the broader governance system. Figure 3 presents the integrated regulatory–HTA framework developed from the thematic synthesis, illustrating how evidence generation, regulatory pathways, HTA processes, pharmacovigilance systems, and financing mechanisms interact to support coherent and evidence-informed policymaking for herbal medicines. In line with national science and technology priorities, herbal medicine should be more explicitly positioned within Indonesia’s research agenda to support coordinated R&D, strengthen translational evidence, and improve system readiness for integrating herbal products into health services.

### Integrated synthesis

Taken together, the descriptive mapping, inductive coding, and deductive alignment provide a comprehensive picture of how evidence functions within Indonesia’s herbal medicine regulatory and HTA ecosystem. Findings point to structural misalignments in which limited evidence maturity undermines regulatory predictability, HTA feasibility, and financing decisions, while fragmented governance restricts system-wide coordination. These challenges are consistent with broader constraints documented in LMIC pharmaceutical systems (Hafner et al., 2020; Nemzoff et al., 2021). The integrated findings serve as the foundation for the evidence-informed regulatory and HTA framework presented in the next section.

## Discussion

The findings of this review offer a comprehensive assessment of the structural, methodological, and governance challenges that underpin Indonesia’s regulatory and HTA landscape for herbal medicines. Drawing on 37 studies, the synthesis identifies three overarching system-level themes misalignment between evidence standards and regulatory requirements, limited integration of HTA into herbal medicine decision-making, and weak post-market surveillance and fragmented governance. Comparable patterns have been documented across other LMICs, where widespread use of herbal and traditional medicines coexists with uneven regulatory maturity, limited HTA capacity, and underdeveloped pharmacovigilance systems. These themes reflect persistent patterns in LMIC pharmaceutical systems and have significant implications for Indonesia’s efforts to develop a predictable, evidence-informed regulatory and coverage pathway for herbal medicines.

### Misalignment between evidence standards and regulatory requirements

A central finding concerns the persistent gap between the evidence generally available for herbal medicines and the standards required by contemporary regulatory authorities. As highlighted by WHO, many LMIC regulators attempt to evaluate herbal products using evidence criteria originally designed for conventional pharmaceuticals, resulting in challenges when evidence remains primarily empirical, heterogeneous, or observational in nature (Saggar et al., 2022). This misalignment weakens regulatory predictability and increases the likelihood of inconsistent pre-market decisions (Saggar et al., 2022). Similar regulatory evidence mismatches have been reported in other LMICs, including India and several ASEAN countries, where legal recognition of traditional medicines is not consistently matched by evidence pipelines capable of supporting regulatory appraisal.

International regulatory agencies, such as the European Medicines Agency, underscore the need for standardised evidence packages including robust clinical evaluations, validated safety data, and rigorous quality documentation to support transparent regulatory decisions (EMA, 2025; Ichim & Booker, 2021). However, studies in this review consistently report shortcomings in herbal product dossiers, including a lack of comparator-controlled studies, variable chemical standardisation, and absence of validated biomarkers factors that complicate evaluation of quality, safety, and efficacy (EMA, 2025; Ichim & Booker, 2021).

For Indonesia, these gaps are amplified by limited local research capacity and inconsistent alignment between regulatory, academic, and industrial stakeholders. Addressing these limitations requires strengthening clinical research infrastructure, improving standard-setting mechanisms, and expanding laboratory capacity in line with WHO guidance on regulatory system strengthening (Suliasih & Mun’im, 2022). Without targeted investment and institutional coordination, similar to challenges observed in other LMICs, regulatory decisions will continue to rely on incomplete or inconsistent evidence, constraining the operationalisation of coherent governance frameworks.

### Limited integration of health technology assessment in herbal medicine policy

The second major theme relates to the limited application of HTA in decision-making for herbal medicines. Although HTA is increasingly recognised as a tool for guiding reimbursement, prioritisation, and coverage decisions, many LMICs encounter methodological and institutional challenges that limit its operationalisation (Ai et al., 2024). The complexity of herbal interventions compounds these challenges: heterogeneous formulations, context-dependent usage patterns, lack of standardised outcomes, and limited economic or utility data reduce the applicability of traditional HTA models (Kwon et al., 2021). Comparable HTA constraints have been reported in other LMICs attempting to integrate traditional medicines into national benefit packages, where methodological rigidity and data scarcity impede decision-making.

As the findings demonstrate, gaps in evidence availability undermine the capacity of HTA bodies to conduct comparative effectiveness or cost-effectiveness assessments. Furthermore, the absence of explicit HTA–regulation linkages contribute to variability between market authorisation decisions, clinical uptake, and financing policies a misalignment also documented in broader evaluations of LMIC HTA systems (Chavarina et al., 2023). These implementation needs mirror reform trajectories in other LMICs, where adaptive HTA approaches are being explored but remain unevenly institutionalised.

In Indonesia, ongoing reforms under the JKN/BPJS scheme create increasing demand for transparent, evidence-informed coverage decisions. For herbal medicines to be considered within national benefit packages, HTA processes must be adapted to accommodate complex interventions and integrate real-world evidence. Methodological flexibility through adaptive modelling, extended uncertainty analyses, and multi-criteria decision analysis is therefore essential to strengthen the relevance and feasibility of HTA for herbal medicines (Chavarina et al., 2023).

### Weak post-market surveillance and fragmented governance

The third theme highlights persistent weaknesses in post-market surveillance and governance structures. The review shows that pharmacovigilance (PV) systems for herbal medicines in Indonesia and many LMICs remain underdeveloped heavily reliant on passive reporting, lacking proactive detection mechanisms, and insufficiently linked to laboratory verification processes (Ekor, 2014). Underreporting of adverse events, combined with limited integration between health facilities, laboratories, and regulatory bodies, constrains the timely identification of safety risks. These challenges are consistent with PV system assessments in other LMICs, where both structural fragmentation and limited operational capacity undermine effective safety surveillance.

Fragmented governance compounds these challenges. Studies report weak coordination among ministries, regulatory authorities, health insurance bodies, and local governments an issue similarly observed in BMJ Global Health analyses of traditional medicine governance across Asia (Sheikh et al., 2017). This fragmentation reduces the coherence of regulatory action and limits the effectiveness of enforcement, particularly in addressing adulteration, contamination, or mislabelling of herbal products. Strengthening coordination between BPOM, the Ministry of Health, BPJS, and research institutions will be critical to improving surveillance systems and ensuring effective safety oversight.

### System-level interdependencies

The interactions identified across evidence generation, regulatory standards, HTA processes, and financing mechanisms demonstrate deep structural interdependencies. Weak clinical and safety evidence constrains regulatory decision-making; limited regulatory clarity undermines HTA feasibility; inadequate HTA integration weakens reimbursement pathways; and fragmented financing mechanisms reduce incentives for research, post-market monitoring, and product quality assurance (Wirtz et al., 2017). These feedback loops generate a cycle of fragmented decision-making an issue consistent with OECD analyses emphasising the need for coordination across evidence, regulation, and financing to strengthen pharmaceutical systems (OECD, 2018).

### Implications for pharmaceutical policy and practice

The findings carry significant implications for Indonesia’s pharmaceutical and traditional medicine policy landscape. Strengthening the regulatory and HTA framework will require:

1. Establishing evidence standards aligned with regulatory expectations.
2. Adapting HTA methodologies to complex herbal interventions.
3. Developing a national pharmacovigilance ecosystem tailored to herbal products.
4. Integrating regulatory, HTA, and financing decisions within a unified policy pathway.
5. Enhancing multisectoral governance, digital traceability, and laboratory capacity (WHO, 2021a).

These implementation priorities are consistent with policy reform agendas in other LMICs and highlight the need for deliberate sequencing, institutional ownership, and sustained investment to translate conceptual frameworks into operational systems.

These steps align with policy recommendations from WHO, EMA, and global HTA networks and provide a pathway for improving regulatory maturity and health system readiness for integrating herbal medicines.

### Strengths and limitations

The review’s strengths include the application of a structured integrative review design, use of WHO-based quality thresholds, and a systems-level synthesis that connects regulatory, HTA, PV, and governance perspectives. These strengths enhance interpretive robustness and policy relevance. However, several limitations should be acknowledged. First, heterogeneity of study designs and evidence types limited cross-country comparability. Second, the scarcity of Indonesia-specific economic evaluations constrained deeper analysis of financing implications. Third, reliance on published and accessible grey literature may have excluded unpublished regulatory experiences or internal policy deliberations. These limitations reflect broader evidence gaps in herbal medicine research globally.

### Future directions

Future research should prioritise standardised clinical evaluation of herbal products (Gagnier et al., 2006), strengthen chemical characterisation to support reproducibility (Heinrich et al., 2018), and progress HTA methodologies suitable for complex interventions (Ai et al., 2024). Policymakers should consider adaptive HTA models, integration of real-world evidence (Nemzoff et al., 2021), and the application of multi-criteria decision analysis to enhance prioritisation transparency (Youngkong et al., 2011). Strengthened collaboration among regulatory bodies, HTA agencies, insurers, and research institutions will be essential for advancing an integrated, evidence-informed policy environment.

### Policy implications

From a policy perspective, the findings of this review underscore the urgency of establishing a coherent and evidence-informed governance pathway for herbal medicines within Indonesia’s health system. Strengthening the alignment between regulatory assessments, HTA processes, pharmacovigilance functions, and financing decisions is essential to enhance transparency, predictability, and public accountability. Such alignment would also support the National Health Insurance (*jaminan kesehatan nasional, JKN*) in making consistent and defensible benefit package decisions, reduce regulatory uncertainty for industry stakeholders, and improve patient safety through more coordinated surveillance systems. Embedding the proposed framework within existing regulatory reform and health financing agendas offers a pragmatic pathway for implementation in Indonesia and other LMICs facing similar systemic constraints.

## Conclusion

This review demonstrates that Indonesia’s regulatory and HTA landscape for herbal medicines is constrained by persistent gaps in evidence maturity, regulatory consistency, HTA feasibility, pharmacovigilance capacity, and multisectoral governance. These system-level misalignments limit the ability of policymakers, regulators, HTA bodies, and health financing institutions to make transparent, predictable, and evidence-informed decisions regarding the authorisation, safety oversight, and potential coverage of herbal products. By synthesising 37 studies across clinical, regulatory, economic, and governance domains, this review offers one of the first integrated syntheses tailored to Indonesia’s health system, emphasising alignment of evidence standards with regulatory pathways, methodological adaptation of HTA for complex herbal interventions, strengthening of pharmacovigilance and laboratory systems, and clearer linkage between regulatory and financing decisions. Advancing this framework will require coordinated action across BPOM, the Ministry of Health, BPJS, and research institutions to establish a coherent, accountable, and evidence-based policy environment capable of supporting safe, effective, and financially responsible integration of herbal medicines into national health services.
